# Impact of microcin J25 on the porcine microbiome in a continuous culture model

**DOI:** 10.3389/fmicb.2022.930392

**Published:** 2022-08-03

**Authors:** Sabrine Naimi, Séverine Zirah, Anna Greppi, Christophe Lacroix, Sylvie Rebuffat, Ismail Fliss

**Affiliations:** ^1^STELA Dairy Research Center, Institute of Nutrition and Functional Foods, Université Laval, Quebec City, QC, Canada; ^2^Laboratoire Molecules of Communication and Adaptation of Microorganisms (MCAM), Sorbonne Université, Muséum national d’Histoire naturelle, Centre National de la Recherche Scientifique, Paris, France; ^3^Laboratory of Food Biotechnology, Institute of Food, Nutrition and Health, ETH Zürich, Zurich, Switzerland

**Keywords:** swine colonic microbiota, PolyFermS model, microcin J25, Illumina MiSeq, LC-MS

## Abstract

The increased prevalence of *Salmonella* spp. resistance in swine spurs the search for alternatives to antibiotics. Microcin J25 (MccJ25), a bacteriocin produced by *Escherichia coli*, is a potent inhibitor of several pathogenic bacteria including *Salmonella enterica*. In this study, we aimed to evaluate *in vitro* the impact of MccJ25 on the composition and the metabolic activity of the swine colonic microbiota. The PolyFermS *in vitro* continuous fermentation model was used here with modified Macfarlane medium to simulate the porcine proximal colon. During 35 days of fermentation, a first-stage reactor containing immobilized swine fecal microbiota fed two second-stage control and test reactors operated in parallel and used to test the effects of MccJ25 on the composition and the metabolic activity of the microbiota. Reuterin, a broad-spectrum antimicrobial compound produced by *Limosilactobacillus reuteri*, a lactic acid bacterium naturally present in the gastro-intestinal tract of human and animals, and the antibiotic rifampicin were tested for comparison. Sequencing of 16S rRNA was performed using the Illumina MiSeq technology to evaluate microbial diversity, and liquid chromatography coupled to mass spectrometry (LC-MS) followed by multivariate analysis was used to assess the bacteriocin/antibiotic degradation products and to monitor changes in the swine colonic microbiota metabolome. The results show that MccJ25 or reuterin treatments only induce subtle changes of both the microbial diversity and the metabolome of the swine colon microbiota, while rifampicin induces significant modification in amino acid levels. Although these findings need being validated *in vivo*, this study affords a first proof of concept for considering MccJ25 as a possible alternative to antibiotics for veterinary and farming applications, taking into account its pathogen-selective and potent inhibitory activity.

## Introduction

Due to continued expansion of international markets, swine production has become one of the fastest growing economic sectors (Kanis et al., 2003; Steinfeld et al., 2006; Lassaletta et al., 2019). In 2019, pork was the second dominant meat produced worldwide, with 110 million tons or 32.6% of total meat production. In spite of this, the swine industry has suffered major losses in recent years, largely due to the increasing incidence of bacterial infections (Abubakar et al., 2017; VanderWaal and Deen, 2018; Foster et al., 2021). Salmonellosis is particularly prevalent in swine, in which it causes acute disease resulting in increased mortality and reduced productivity (Bonardi, 2017; Yuan et al., 2018; Bearson, 2021). Indeed, swine is a major reservoir for *Salmonella enterica* which has been associated to diarrhea in pig farming. Although swine diarrhea, especially in young pigs, originates from dysfunction of the small intestine, the other major cause is inflammation of the colon, often referred to as colitis (Panah et al., 2021). Moreover, many *Salmonella* serovars have been shown to be associated with chronic ulcerative colitis or necrotizing enterocolitis in pigs. Thus, controlling *Salmonella* infections in pig farming, especially in industrialized countries, has been achieved in large part through the use of antibiotics (Cromwell, 2002).

Ever since their commercialization began in the 1950’s, antibiotics have been used widely in human and veterinary medicine as well as in livestock production in order to treat bacterial diseases and promote animal growth (Oliveira et al., 2020; Hosain et al., 2021). Their use as growth promoters in swine production is commonplace and has contributed substantially to improved livestock productivity and reduced production costs (Lekagul et al., 2019). However, their overuse has led to the emergence of multi-drug resistant bacteria, both commensal and pathogenic, which can be transmitted to humans through food consumption, contact with animals, or the release of excreta into the environment (Barton, 2014). This has raised public concern and has led several countries to ban the use of antibiotics as growth promoters in animal feeds, as the European Union did in 2006 (Mathew et al., 2007). Some other countries, including the United States, Canada, Mexico, Japan, Hong Kong, China and India have limited the use of antimicrobials in feed and tried to reduce antibiotics in food animal production (Salim et al., 2018). Effective replacements for conventional antibiotics are now urgently needed in order to maintain swineherd health and productivity. Among the most promising alternatives proposed so far are natural antimicrobial peptides (AMPs) produced by bacteria, called bacteriocins (Sang and Blecha, 2008; Cotter et al., 2013; Cavera et al., 2015; Vieco-Saiz et al., 2019; Telhig et al., 2020).

Bacteriocins are defined as bacterially produced antimicrobial peptides of molecular mass usually less than 10 kDa (Riley and Wertz, 2002; Cotter, 2005). Unlike commonly used broad spectrum antibiotics, bacteriocins are translated from mRNA and have a narrow spectrum of activity, inhibiting strains of the same or closely related species at nanomolar concentrations (Cleveland et al., 2001; Chikindas et al., 2018; Soltani et al., 2021). They have been categorized in different classes based on their structural characteristics, and are listed in an open-access database called BACTIBASE (Hammami et al., 2010). The majority of bacteriocins studied so far are produced by Gram-positive bacteria and most often lactic acid bacteria, which are widely used in the food industry (Arthur et al., 2014). However, numerous structurally diverse Gram-negative bacteriocins are also described (Telhig et al., 2020; Calcuttawala et al., 2021). Bacteriocins find applications in a wide range of sectors including agri-food, and the human and veterinary medicine fields (Stahl et al., 2004; Cutler et al., 2007; Soltani et al., 2021). Among Gram-negative bacteriocins, some colicins that are proteinaceous bacteriocins produced by *Escherichia coli*, have been studied for potential applications in the treatment of various human and animal infectious diseases (van Winsen et al., 2001; Stahl et al., 2004; Cutler et al., 2007) and as inhibitors of enteric pathogens in animals, including swine. However, despite such studies showing that the inhibitory activity of bacteriocins and their impact on the colon microbiota are real albeit not yet well understood, there are currently very few data on the impact of bacteriocins on the overall metabolic profile of the colon microbiota (Benítez-Chao et al., 2021).

Microcin J25 (MccJ25) is one of the most studied Gram-negative bacteriocins. This 21-amino-acid posttranslationally modified peptide produced by *E. coli* is a potent inhibitor of *Enterobacteriaceae* including *Salmonella* (Rintoul et al., 2001). It is a typical lasso peptide (Arnison et al., 2013; Wang and Zhang, 2018) which is characterized by an 8-residue macrolactam ring that is closed by an isopeptide bond between the N-terminus and the side chain carboxylate of a glutamate at position 8. The ring is threaded by the resulting tail that is locked inside by two bulky amino acid side chains located above and below the ring, thus forming a loop. This highly compact structure confers MccJ25 remarkable stability to denaturing conditions, high temperatures, extreme pHs and certain proteases (Rebuffat et al., 2004; Vincent and Morero, 2009; Telhig et al., 2020). Our group has previously demonstrated the inhibitory activity of MccJ25 against *Salmonella* Newport under conditions simulating those of the swine proximal colon using the Polyfermentor intestinal model (PolyFermS) (Naimi et al., 2020). Indeed, this inhibitory activity remained potent over 24 h of continuous culture and was superior to those of two other well-known antimicrobial compounds, a broad-spectrum antibiotic rifampicin and reuterin, a biocide produced by a lactic acid bacterium (Naimi et al., 2020). Moreover, the inhibitory activity of MccJ25 was shown to be correlated with its high stability that has been attributed to its unique lasso structure (Salomon and Farías, 1992; Rosengren et al., 2003) and its bioavailability. Such characteristics tightly fit with the present requirement of antibiotics having high efficiency, but devoid of collateral damages on the commensal bacteria in the gut microbiota, which result in dysbiosis and related diseases (Melander et al., 2018). Such interesting features of MccJ25 make it appealing for a possible use as a natural alternative to antibiotics in pig feed. Indeed, a diet supplemented with MccJ25 (purity above 99%) has been shown to improve growth performance, and attenuate diarrhea and systematic inflammation of weaned pigs (Yu et al., 2017). These properties were accompanied with a decrease in *E. coli* and an increase in *Lactobacillus* and *Bifidobacterium* species, which improve short-chain fatty acid (SCFA) content. However, the other bacterial families and genera were not examined.

In the present study, we evaluated the impact of MccJ25 on the composition and the metabolic activity of the swine colonic microbiota *in vitro*, using the PolyFermS continuous fermentation model to simulate the swine proximal colon (Tanner et al., 2014; Naimi et al., 2020). The results obtained with MccJ25 were compared to those of rifampicin, a broad-spectrum conventional antibiotic, and reuterin, an antimicrobial compound produced by *Limosilactobacillus reuteri*, which is a lactic acid bacterium naturally present in the gastro-intestinal tract of human and animals and is used as a probiotic (Cleusix et al., 2007, 2008; Asare et al., 2020; Liang et al., 2021). Furthermore, we examined the behavior of the three antibacterial compounds in the swine colonic conditions by studying and identifying their degradation products.

## Materials and methods

### Bacterial strains

*Escherichia coli* MC4100 carrying the plasmid pTUC202 was obtained from the MCAM laboratory collection (Muséum national d’Histoire naturelle, Paris, France) and used for MccJ25 production (Solbiati et al., 1999). *L. reuteri* ATCC 53608 purchased from American Type Culture Collection (ATCC, Manassas, VA, United States) was used for the production of reuterin. *Salmonella enterica* subsp. enterica serovar Newport ATCC 6962 (hereinafter called *Salmonella* Newport) was purchased from Microbiologics Inc. (St. Cloud, Minnesota, United States). Both *E. coli* MC4100 and *Salmonella* Newport were cultured overnight at 37°C in Luria-Bertani medium, LB (tryptone 10 g/L, yeast extract 5 g/L, sodium chloride 10 g/L; Difco, Sparks, MD, United States; batch 244620), under aerobic condition (Solbiati et al., 1999; Naimi et al., 2020). *L. reuteri* ATCC 53608 was inoculated (1% v/v) and cultured anaerobically at 37°C for 16 h in MRS broth medium (Nutri-Bact; catalog number QB-39-2285) supplemented with 20 mM glycerol (Doleyres et al., 2005).

### Antimicrobial compounds

MccJ25 was produced from a culture supernatant of *E. coli* MC4100 that harbors the plasmid pTUC202 following the protocol previously described by Ducasse et al. (2012). In this study, 40 liters of broth culture of MccJ25-producing strain were used as detailed previously (Naimi et al., 2020). Briefly, minimal medium (M63) inoculated with a preculture of strain MC4100 in LB broth was incubated overnight at 37°C in a pilot fermenter (BIOSTAT^®^ C plus, Göttingen, Germany). The culture was centrifuged as described previously and the supernatant was run through a Sep-Pak C18 35 cc cartridge (Waters, Milford, United States). MccJ25 was eluted with acetonitrile/water (30% v/v) containing 0.1% HCl then further purified by reverse-phase HPLC on a preparative C18 column at a flow rate of 10 mL/min using a 25–100% linear gradient of filtered acetonitrile/5 mM HCl in ultra-pure water with absorbance measurement at 214 nm for peptide detection. The purified MccJ25 (purity = 98−99%, HPLC) was quantified by RP-HPLC using an analytical C18 column. The purified MccJ25 was then stored at 4°C until use.

Reuterin (3-hydroxypropanal) was produced using a culture of *L. reuteri* ATCC 53608 in MRS medium containing 20 mM glycerol according to a method described previously (Vimont et al., 2019). The used protocol was detailed in our previous work (Naimi et al., 2020). Briefly, the bacterial culture was first centrifuged, and the cells were washed with 0.1 M potassium phosphate buffer (pH 7.0), centrifuged again, re-suspended in glycerol solution, held at room temperature for 45 min under anaerobic conditions and centrifuged. The filtrate was lyophilized and reuterin was purified on a silica gel 60 preparative chromatography column (2.8 × 35 cm, Bio-Rad Econo-Column) with acetone:ethyl acetate (2:1) as eluent at a flow rate of 5 mL/min. Reuterin was quantified using a colorimetric method described previously by Lüthi-Peng et al. (2002). The obtained solution containing purified reuterin (purity = 95%, HPLC) was stored at −20°C until use.

Rifampicin (purity ≥ 97%, HPLC) was purchased from Sigma-Aldrich (St. Louis, MO, United States) and stored at −20°C until use.

### PolyFermS fermentation model of the swine colon

#### Immobilization of fecal microbiota and fermentation medium

A fecal sample from a healthy adult swine raised under farm conditions and not given any antibiotic during the previous three months was collected in a sterile 50 mL Falcon tube. Anaerobiosis was maintained in a Gas-Pak anaerobic jar (Oxoid, Thermo Fisher Scientific) during transport to the laboratory and until the immobilization procedure (Cinquin et al., 2004). As described previously, about 20 g were suspended in 80 mL of 0.1% peptone water (Difco Laboratories) containing 0.05% L-cysteine-HCl (Sigma-Aldrich, St. Louis, MO, United States) at 37°C and homogenized for 5 min at 200 rpm using a Stomacher (Seward model 400, Norfolk, United Kingdom) (Naimi et al., 2020). The liquid portion was recovered using a serological pipette and centrifuged for 1 min at 700 x g to remove large particles. The immobilization procedure was begun in an anaerobic chamber (model 1025, Forma Scientific, Marietta, OH, United States) by mixing 20 mL of supernatant into 1 L of a solution of 2.5% w/v gellan, 0.25% w/v xanthan and 0.2% w/v sodium citrate (Cinquin et al., 2004). After inoculation, the polymer solution was stirred into freshly autoclaved (15 min at 121°C) hydrophobic phase (commercial canola oil) at 43°C to obtain a suspension of aqueous droplets in oil. Gel beads were formed by cooling the suspension. After separation and washing, beads were hardened by soaking for 30 min in a 0.1 M CaCl_2_ solution. Gel beads with diameters in the 1-2 mm range were selected by wet sieving and about 75 g were transferred to the inoculum reactor (BIOSTAT^®^ Q plus, Sartorius AG, 79 Göttingen, Germany) containing 250 mL of fresh fermentation medium.

The modified Macfarlane medium used in this study simulates the conditions in the swine proximal colon as described previously (Naimi et al., 2020). The medium was autoclaved at 121°C for 20 min and then stored at 4°C until use. Solutions of L-cysteine-HCl monohydrate (5%) and vitamins as described previously by Michel et al. (1998) were filter-sterilized (0.22 μm, Millipore) and added separately to the sterilized medium (16 mL/L and 0.5 mL/L, respectively). All components of the nutrient medium were purchased from Sigma-Aldrich (St. Louis, MO, United States).

#### Fermentation procedure and treatments

**FIGURE 1:**
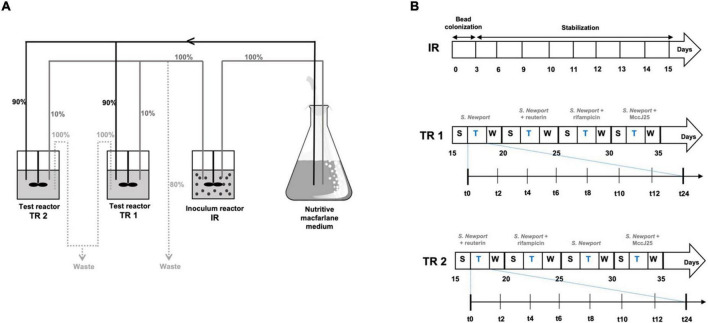
Experimental set-up of the continuous fermentation experiment. **(A)** Overview of the PolyFermS model composed of one inoculum reactor and two test reactors and experimental plan used: relative flow rates are indicated in%. IR: inoculum reactor containing pig fecal bacteria immobilized in gel beads (30% v/v); TR1, TR2: test bioreactors; S: stabilization period; T: treatment period; W: wash period. **(B)** Experimental treatment and samples collection schedule.

The *in vitro* fermentation model of the swine colon has been described elsewhere in detail (Tanner et al., 2014) and in our previous study (Naimi et al., 2020). Briefly, the PolyFermS model used for this study consisted of a two-stage system comprising a total of three glass reactors (BIOSTAT^®^ Qplus, Sartorius AG, Göttingen, Germany) as shown in Figure 1, which were controlled at 38°C and pH 6.0 with anaerobic conditions maintained by continuous flow of pure CO_2_ through the medium as described by Fernandez et al. (2016). Seeded with 30% (v/v) swine fecal beads, the inoculum reactor IR (useful volume of 250 mL) was fed fresh nutrient medium continuously (Figure 1A). Effluent from IR was used to continuously inoculate at 10% (v/v) the test reactors TR1 and TR2, which also received 90% (v/v) fresh medium (Figure 1B). The feed rate of the three reactors controlled using peristaltic pumps (model 120U, Watson-Marlow, Falmouth, Cornwall, United Kingdom) was set at 27 mL/h for a mean retention time in IR and TRs of 9 h. For the first 3 days, the IR was run in repeated-batch mode, the medium replaced every 12 h with fresh medium, in order to colonize fecal bead. Continuous feeding was then started, followed by 12 days of stabilization before connection to TR1 and TR2. Continuous culture was split into three periods: stabilization, treatment and wash. Conditions were kept constant in IR to assess the temporal stability of the system without any manipulation during the entire fermentation, while TR1 and TR2 were subjected to treatments for various periods. Between each period, culture was flushed from TR1 and TR2 as described previously (Tanner et al., 2014). TR1 and TR2 were subjected to a washing procedure with 10% chlorine solution to decontaminate and remove any historical effect of the previous periods. Briefly, TR1 and TR2 were disconnected from the IR, the entire medium was removed and reactors were filled with 10% freshly prepared chlorine solution. After stirring for 1 h, the reactors were rinsed twice by adding sterile distilled water and stirring for another hour. After complete removal of water and chlorine residues, reactors were filled with sterile fresh nutritive medium and reconnected to the IR. After each flush, they were stabilized for 3 days until a steady state was reached before starting the next treatment period.

Performed simultaneously in TR1 and TR2, the four treatments consisted of adding *Salmonella* Newport alone (Salmo) at an initial concentration of 10^7^ cfu/mL to each reactor (Figure 1C), followed by adding *Salmonella* Newport at this concentration along with each tested antimicrobial: 0.4 mM MccJ25 (J25), 4 mM reuterin (Reut) or 0.6 mM rifampicin (Rifa). These concentrations are based on preliminary assays performed in our previous study (Naimi et al., 2020). For each treatment, samples of test reactor effluent were collected at 0, 2, 4, 6, 8, 10, 12, 24, 48, and 72 h for analysis and stored at −80°C.

### Propidium-monoazide-coupled quantitative polymerase chain reaction

#### Treatment with propidium monoazide

The inhibitory activity of MccJ25, reuterin and rifampicin against *Salmonella* Newport and the major bacterial species constituting the swine colonic microbiota were quantified using propidium-monoazide-coupled quantitative polymerase chain reaction (PMA-qPCR). Samples collected from the reactors were treated with propidium monoazide (PMA dye, Biotium, Inc., Hayward, CA, United States) for viable bacteria counts as described previously (Fernandez et al., 2016). Briefly, 2.5 μL of 20 mM PMA solution (1 mg of PMA dissolved in 91 μL of 20% dimethylsulfoxide, stored at −20°C) were added to each 1 mL sample collected from the reactors to obtain a final concentration of 50 μM. Samples were kept for 5 min at room temperature in the dark with occasional vortex mixing, then placed for 5 min on ice 20 cm below a 500 W halogen lamp. Samples were then frozen and stored at −80°C until DNA extraction.

#### DNA extraction

DNA was extracted from samples of fermentation broth using the PowerSoil™ DNA Isolation Kit (MO BIO Laboratories, CA, United States). Samples were thawed and centrifuged for 10 min at 12,000 × *g* and the pellets were washed twice in Tris-EDTA buffer (10 mM Tris-HCl, 1 mM EDTA). Subsequent steps were performed following the PowerSoil kit instructions.

#### Quantitative polymerase chain reaction analysis

The quantitative PCR (qPCR) was performed in MicroAmp^®^ Fast Optical 96-Well Reaction Plates with Barcode (Life Technologies Inc., Burlington, ON, Canada) on an ABI 7500 Real-Time PCR System (Applied Biosystems, Streetsville, ON, Canada). Each sample was analyzed in duplicate. Extracted DNA was diluted 1/10 (v/v) in DNase-free water (Invitrogen) and placed in the microplate wells (5 μL of diluted extract per well). Each well also contained 12.5 μL of Fast SYBR^®^ Green Master Mix (Applied Biosystems, Burlington, ON, Canada), 1 μL of each primer, forward and reverse, at a final concentration of 5 μM (Sigma-Aldrich, St. Louis, MO, United States) and 5.5 μL of DNase-free water. The primers used in this study are given in Supporting information (Supplementary Table 1). The amplification program consisted of an initial denaturation at 95°C for 10 min, followed by 40 cycles of amplification at 95°C for 15 s and 60°C for 1 min (Fernandez et al., 2016). Standard curves were obtained for the quantified bacterial species by extracting DNA from reference strain pure culture re-suspended in 1/10 diluted Macfarlane broth and treated with PMA. The cultures were also enumerated as colonies forming units (cfu) on agar medium. DNA extracted from each strain was then serially diluted 10-fold to obtain equivalent from 10^9^ to 10^2^ cfu mL^–1^. Standard curves were generated from plots of threshold cycle (Ct) versus bacterial count (cfu mL^–1^). The bacterial counts (cfu mL^–1^) were interpolated from the averaged standard curves.

### Microbial community analysis

#### 16S rRNA amplicon sequencing

The microbial community at different time points along the fermentation was analyzed by Illumina next-generation 16S rRNA gene amplicon sequencing. Taxonomic analysis were assessed by sequencing the bacterial 16S rRNA gene in the V3-V4 region using the amplification primers 341F (5′-CCTACGGGNGGCWGCAG-3′) and 805R (5′-GACTACHVGGGTATCTAATCC-3′) adapted to incorporate the transposon-based Illumina Nextera adapters (Illumina, United States) and a sample barcode sequence allowing multiplexed sequencing (García-López et al., 2020). High-throughput sequencing was performed at the Institute for Integrative Systems Biology (Université Laval, Québec, Canada) on a MiSeq platform using 2 × 300 bp paired-end sequencing (Illumina, United States). The raw sequence data have been submitted to European Nucleotide Archive (ENA) database with accession number PRJEB52762.

#### Sequence analysis

Raw data were processed using the DADA2 R package (version 1.14.1) (Callahan et al., 2016) to obtain exact amplicon sequence variants (ASVs) using the metabaRpipe R package^1^. Briefly, forward and reverse reads were truncated after 260 and 250 nucleotides, respectively. After truncation, reads with expected error rates higher than three and four for forward and reverse reads, respectively, were removed. After filtering, error rate learning, ASV inference and denoising, reads were merged with a minimum overlap of 15 nucleotides. Chimeric sequences were identified and removed. Taxonomy was assigned to ASVs using DADA2 against SILVA database (v138.1) (Glöckner et al., 2017). Phylogenetic relatedness of ASVs, alpha diversity (Observed and Shannon indexes) and beta diversity analyses (Bray Curtis similarity matrix) were performed using the phyloseq R package (McMurdie and Holmes, 2013) and the DivComAnalyses packages^2^.

### Metabolomic analysis

#### Samples preparation

Samples were prepared using the quenching method described previously (Wellerdiek et al., 2009). Briefly, 1 mL of fermentation broth was drawn into a syringe containing 4 mL of aqueous methanol (60%, v/v) at −40°C and then centrifuged at 4,000 × *g* for 5 min at 4°C. The supernatant was discarded and only cell pellet was kept for the analysis of intracellular metabolites. Collected cell pellet was kept in a tube on ice and then re-suspended in methanol, chloroform and water (1:1:0.9 v/v/v) with vortex mixing after adding each solvent and then centrifuged at 8,000 × g for 10 min at 4°C. A 70 μL aliquot of the upper water layer was collected in an Eppendorf tube and stored at −80°C until LC/MS analysis (50 μL of thawed sample in the glass vial).

#### Liquid chromatography coupled to mass spectrometry analysis

Five micro liters of each resuspended sample were analyzed by ultra-high-performance liquid chromatography system (Ultimate 3000 RSLC, Thermo Scientific) connected to a high-resolution electrospray ionization – quadrupole – time of flight (ESI-Q-TOF) mass spectrometer (Maxis II ETD, Bruker Daltonics). Separation was achieved on an Acclaim RSLC Polar Advantage II column (2.2 μm, 2.1 × 100 mm, Thermo Scientific) at a flow rate of 300 μL/min, using the following gradient of solvent A (ultra-pure water/0.1% formic acid) and solvent B (HPLC-MS grade acetonitrile/0.08% formic acid) over a total run time of 17.5 min: 5 min at 0% B followed by a linear increase from 0 to 60% B for 12 min, linear increase to 100% B for 0.2 min, decrease to 0% B for 0.5 min. The MS spectra were acquired in positive ion mode in the mass range *m/z* 60 – 1300. The source parameters were as follows: nebulizer gas 35 psi, dry gas 8 L/min, capillary voltage 3500 V, end plate offset 500 V, temperature 200°C. The ESI-Q-TOF instrument was externally calibrated before each run using a sodium formate solution consisting of 10 mM sodium hydroxide in isopropanol/0.2% formic acid (1:1, v/v). A quality control (QC) consisting of a mix of all samples and a blank (injection of water) were recorded every 10 samples to monitor separation quality and absence of cross-contaminations. Data-dependent LC-MS/MS experiments were also conducted, using collision dissociation of 1 +, 2 +, and 3 + ions preferentially, using a 3 s cycle time for MS scans at 2 Hz, and 1 Hz to 3 Hz MS/MS scans depending on intensity.

The high-resolution LC-MS data were converted to netCDF format, while the LC-MS/MS data were converted to mgf format, using Data Analysis 4.4 software (Bruker Daltonics). The netCDF converted LC-MS data were analyzed using the freely available R environment version R-4.0.4.^3^ Xcms package was used for peak detection and peak matching, on the 0.5 – 13.4 min range. The parameters were as follows: centwave peak detection method, maximal tolerated *m/z* deviation in consecutive scans: 10 ppm, prefilter: 5 peaks with intensity ≥ 5000, chromatographic peakwidth: between 5 and 30 s, signal to noise ratio cutoff 0, minimum difference in *m/z* for peaks with overlapping retention time -0.005. The multivariate matrix generated (197 samples × 5095 peaks) was submitted to principal component analysis (PCA) using mixOmics package (Lê Cao et al., 2009). The mgf converted LC-MS/MS data were used to generate a molecular network using the online GNPS workflow (Yang et al., 2013; Wang et al., 2016), using the following set-up: parent ion mass tolerance of 0.05 Da, fragment ion mass tolerance 0.05 Da, cluster minimal size 5, minimum matched peaks 4, and minimum cosine similarity score 0.5. Resulting networks were visualized using Cytoscape 3.7.1.

### Statistical analysis

The rarefied ASV table was used to calculate richness and diversity indices. Significant differences in alpha diversity of each treatment group at each time point were assessed by Wilcox test, while differences in beta diversity were checked by PERMANOVA (p-value 0.05) using R.

## Results

### Impact of MccJ25, reuterin and rifampicin on bacterial groups representing the colonic microbiota

Based on analysis by PMA-qPCR, counts of specific bacterial groups in both test reactors TR1 and TR2 remained relatively stable in overall composition for the first 12 h following the addition of *Salmonella* Newport plus antimicrobial agents as shown in Supplementary Figure 1. However, rifampicin was shown to induce an effect on total cell and *Bacteroides* counts resulting in a reduction of approximately 1 log after 12 h, followed by regrowth of these bacterial groups in addition to *Lactobacillaceae* until the end of the fermentation (Supplementary Figure 1C). A similar effect, especially on *Lactobacillaceae* counts, was observed following the treatment with *Salmonella* Newport alone after 12 h of fermentation as shown in Supplementary Figure 1A. In contrast, both reuterin (Supplementary Figure 1B) and MccJ25 (Supplementary Figure 1D) did not induce significant impact on total bacterial counts.

### Impact of MccJ25, reuterin and rifampicin on the porcine intestinal microbiota diversity

**FIGURE 2:**
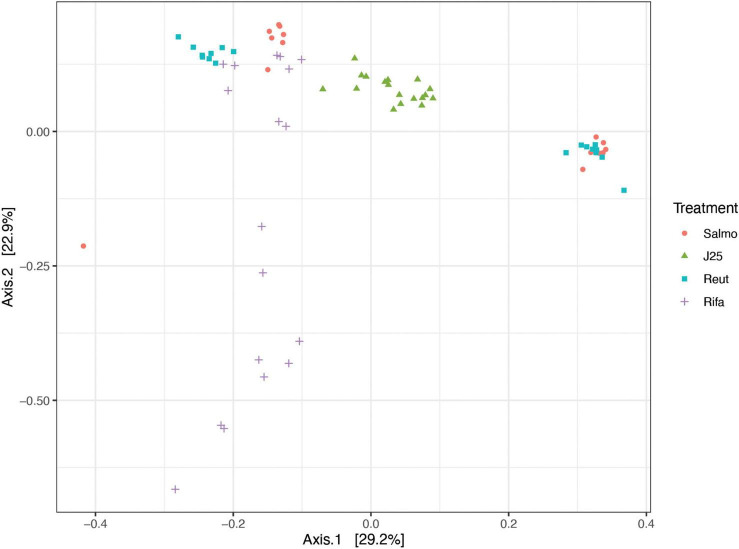
Principal coordinate analysis (PCoA) of porcine microbiota after inoculation of *Salmonella* Newport alone (Salmo) and in combination with MccJ25 (J25), reuterin (Reut) or rifampicin (Rifa) *in vitro*, based on Bray-Curtis similarity matrix from all time points from the two independent experiments. Dots are colored by treatment.

The impact of tested antimicrobials on microbiota composition were evaluated by sequencing the 16S rRNA gene along time. Data were first assessed *via* the use of principal coordinate analysis (PCoA) of the Bray Curtis similarity matrix from all samples in the two replicates F1 and F2 along time (Figure 2) and also in samples separated by each time point (Supplementary Figure 3). No significant changes in microbial composition were detected by *Salmonella* alone or reuterin treatment groups, suggesting no effect of those treatments on the overall microbiota composition profile measured over 24 h fermentation. On the other hand, significant differences (p value 0.001) were detected upon MccJ25 and rifampicin, as compared to *Salmonella* alone and also in rifampicin and reuterin, as compared to MccJ25.

**FIGURE 3:**
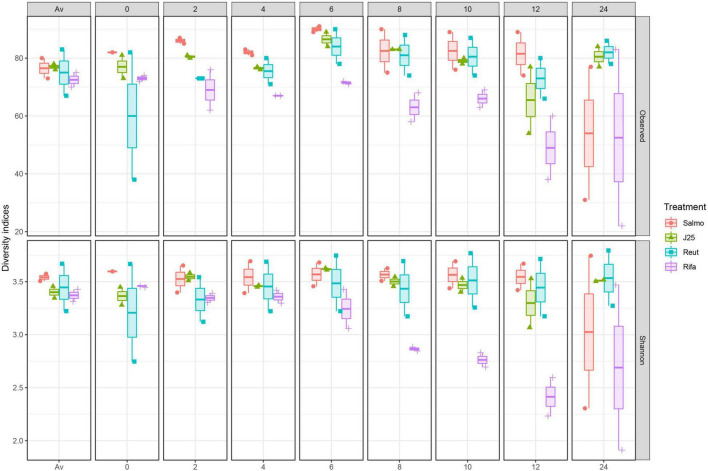
Impact of *Salmonella* Newport alone (Salmo) and in combination with MccJ25 (J25), reuterin (Reut) or rifampicin (Rifa) on the porcine intestinal microbiota *in vitro* as measured by 16S rRNA gene amplicon sequencing. Alpha diversity measured by Shannon index (Shannon) and number of Observed ASVs (Observed) at each fermentation day: Av (before treatment), 0, 2, 4, 6, 8, 10, 12, and 24. Per each treatment and time point, two independent replicates are shown.

The impact of the treatments on the porcine intestinal microbiota was also investigated by alpha diversity, using Shannon index and the number of observed ASVs (Figure 3). The results demonstrated a significant decrease in alpha diversity following rifampicin treatment at the 8-h time point until the end of the fermentation which confirm the impact of rifampicin on microbiota composition. An drop in alpha diversity was also observed in *Salmonella* alone treatment group only at the end of the colonic fermentation, however, it did not appear to be significant. In contrast, MccJ25 and reuterin treatment groups did not induce a remarkable changes in alpha diversity during the fermentation comparing to rifamicin and *Salmonella* treatments.

**FIGURE 4:**
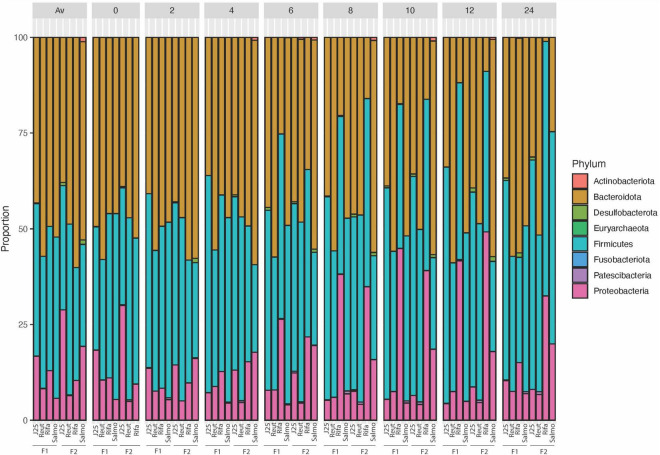
16S rRNA gene-based microbial community structure of porcine microbiota after inoculation of *Salmonella* Newport alone (Salmo) and in combination with MccJ25 (J25), reuterin (Reut) or rifampicin (Rifa). The relative abundance of amplicon sequence variants ASV (%) are represented at the phylum level and samples are grouped at each time point for two independent fermentations (F1 and F2).

**FIGURE 5:**
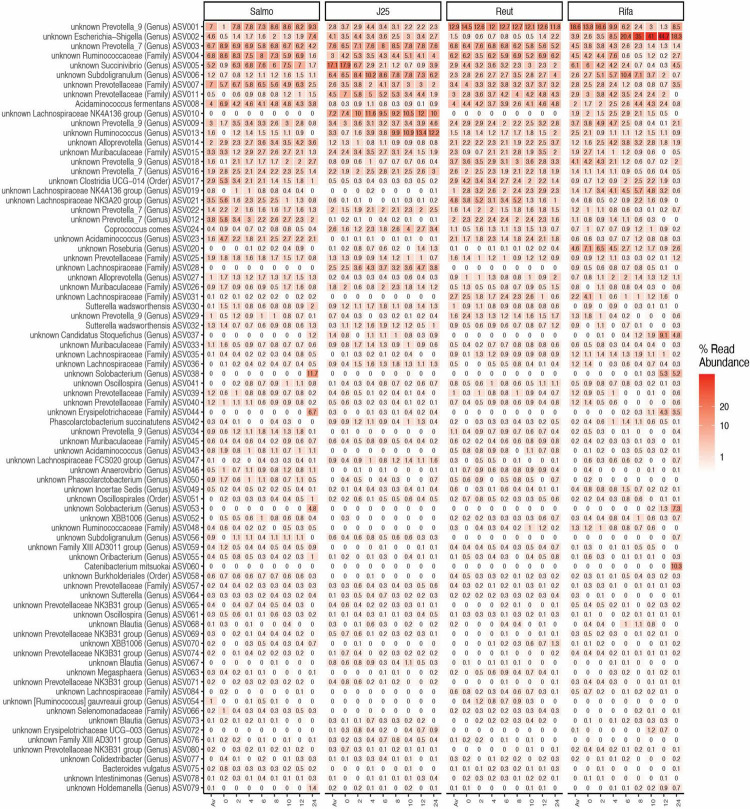
Microbial community structure of porcine intestinal microbiota after inoculation of *Salmonella* Newport alone (Salmo) and in combination with MccJ25 (J25), reuterin (Reut) or rifampicin (Rifa) on the porcine intestinal microbiota *in vitro*. Microbial composition measured by 16S rRNA gene amplicon sequencing at different days: Av (before treatment), 0, 2, 4, 6, 8, 10, 12, 24) represented by relative abundance (%) at the highest-level taxonomy. Values are the average of two biological replicates from two independent fermentations. The most abundant 80 ASVs shared in all treatments are represented.

The impact of tested antimicrobials on the porcine intestinal microbiota composition was further evaluated by taxonomic analysis at the phylum level, which revealed that rifampicin treatment induced an increase of the relative abundance of Proteobacteria and Firmicutes phyla specially from the 6-h time point in both test reactors F1 and F2 (Figure 4). In addition, *Escherichia-Shigella* (ASV002) were significantly enriched by rifampicin from the 8-h time point (exceeding more than 44% in relative abundance at 12 h) (Figure 5). Overall, these data indicate that rifampicin significantly impacted microbiota composition as compared to the other tested antimicrobials in the PolyFermS model and this can be envisioned to impact the metabolic activity of the intestinal microbiota as well.

### Liquid chromatography coupled to mass spectrometry data analysis and metabolomic profiling

**FIGURE 6:**
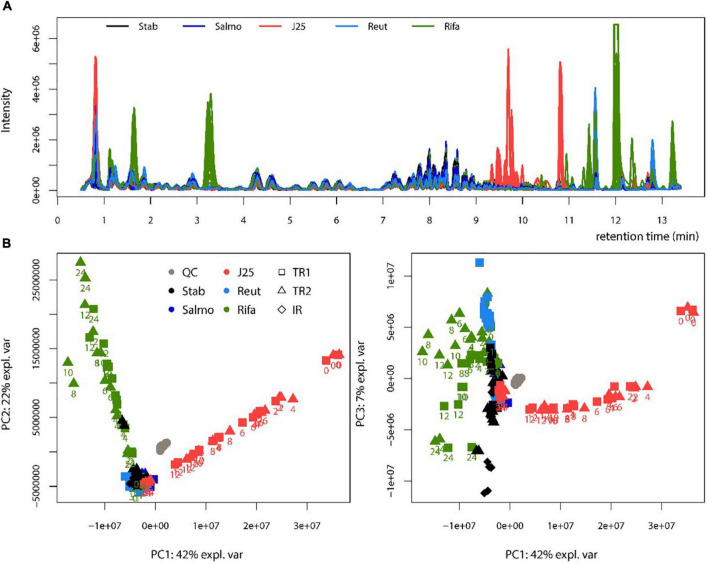
Metabolomic profiling of swine colonic microbiota in the PolyFermS model. **(A)** Base peak chromatograms obtained by LC-MS for each treatment. Stab: stabilization, Salmo: *Salmonella* Newport treatment, J25: *Salmonella* Newport + 0.4 mM MccJ25 treatment, Reut: *Salmonella* Newport + 4 mM reuterin treatment, Rifa: *Salmonella* Newport + 0.6 mM rifampicin treatment; **(B)** Score plot of the PCA obtained from LC-MS data (left: PC1 versus PC2, right: PC1 versus PC3). The reactors sampled (test reactors TR1 and TR2 and inoculum reactor IR) are shown with symbols. The collection times after Rifa and J25 treatments are written below the symbols. QC: control quality.

**FIGURE 7:**
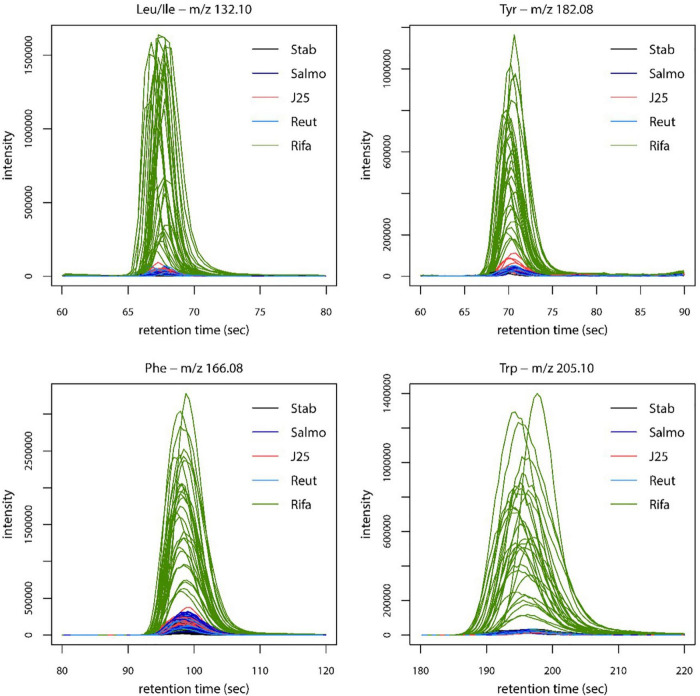
Extracted ion chromatograms representing the relative intensity of Leu/Ile, Tyr, Phe and Trp amino acids in swine microbiota metabolome extracts, for each treatment. Stab: stabilization, Salmo: *Salmonella* Newport treatment, J25: *Salmonella* Newport + 0.4 mM MccJ25 treatment, Reut: *Salmonella* Newport + 4 mM reuterin treatment, Rifa: *Salmonella* Newport + 0.6 mM rifampicin treatment.

The fermentation broth pellet extracts were analyzed by LC-MS in order to evaluate the impact of MccJ25, reuterin and rifampicin on the metabolome of the swine colonic microbiota. The LC-MS profiles and corresponding PCA scores are shown in Figure 6. MccJ25 and rifampicin treatments showed the most important effect on the metabolomics profiles, but the effect of MccJ25 decreased with time while that of rifampicin increased (Figure 6B). The particular profiles obtained for both treatments are related in part to the contribution of MS signals associated to the exogenous compounds added (MccJ25 or rifampicin) and their degradation products. The degradation products could be revealed by molecular networking (Supplementary Figure 3), as already reported for MccJ25 in an *in vitro* digestion model (Naimi et al., 2018). The singly hydrolyzed MccJ25 peptides could be identified unambiguously from MS/MS data (Supplementary Figures 4, 5). The main degradation products of MccJ25 and rifampicin in swine colonic conditions could thus be characterized (Supplementary Tables 2, 3). After 24 h treatment, the metabolomics profiles for MccJ25-treated microbiota were close to that before treatment. By contrast, for rifampicin, significant effects on the swine colonic microbiota metabolome were observed, which increased with time. A major trend observed was an increase in the levels of Leu/Ile, Phe, Tyr and Trp amino acids (Figure 7).

## Discussion

The widespread use of antibiotics in livestock production has led to the emergence of antibiotic-multiresistant bacteria, which can infect humans *via* the food chain. The use of antibiotics as growth promoters has been banned outright in several industrialized countries, which has increased the urgency of the search for alternatives. Researchers began decades ago to propose bacteriocins as substitutes. In addition to their natural origin, being mostly indigenous to the gut microbiota, and their potent inhibitory activity, bacteriocins are most often endowed with a narrow spectrum of antibacterial activity. This prevents causing a disequilibrium of the colonic microbiota and prevent dysbiosis-related diseases. Thus, this is a characteristic that makes narrow-spectrum pathogen-specific antimicrobial agents particularly sought nowadays (Melander et al., 2018). However, few studies have brought clear evidence that bacteriocins inhibit bacterial pathogens under the conditions encountered in the intestines of farm animals. Indeed, MccJ25, which is produced by bacteria of the family *Enterobacteriaceae* and contributes to competition in the gut has a narrow spectrum of antibacterial activity. It is reportedly bactericidal to Gram-negative species including *E. coli*, *Salmonella* and *Shigella* (Rintoul et al., 2001; Yu et al., 2022). Highly purified MccJ25 was recently shown to exert efficient bactericidal activity *in vitro* against clinically relevant veterinary multidrug-resistant strains and to decrease inflammation in a mouse *E. coli* infection model (Yu et al., 2022).

In a previous study using a medium mimicking conditions of the swine colon, we demonstrated that MccJ25 induced a significantly stronger inhibition of the growth of *Salmonella* Newport, selected as an infection model, than two other antimicrobial agents reuterin and rifampicin, used for comparison (Naimi et al., 2020). Rifampicin was selected as a broad-spectrum conventional antibiotic, and reuterin as an antimicrobial compound produced by a lactic acid bacterial strain naturally present in mammalian gut. Here, we used the PolyfermS *in vitro* model, a dynamic *in vitro* model reproducing conditions of the swine colon, to study the impact of MccJ25 on the composition, diversity, stability and metabolic activity of the porcine colonic microbiota and to compare this activity to those of reuterin and rifampicin. Furthermore, a state-of-the-art chromatographic method was optimized for evaluating their impact on the microbiota metabolic activity.

In the lead-up to this study, a stable bacterial population representative of the porcine colonic microbiota was reproduced *in vitro* using the PolyFermS model developed previously by Tanner et al. (2014). This model allows reproducible testing of different treatments in parallel reactors, thus providing a built-in control inoculated with the same microbiota (Naimi et al., 2020). Our results showed first that the bacterial community remained relatively stable following the addition of *Salmonella* in the PolyFermS model. Indeed, introduction of a pathogen in a stabilized microbiota does not always result systematically in a strong modification of the global microbiota composition, as previously observed with *Salmonella enterica* subsp. *enterica* serovar Typhimurium N-15 using the PolyFermS *in vitro* continuous fermentation model simulating the swine proximal colon. This may be different *in vivo*. Indeed, the presence of pathogen may induce translocation an inflammation leading to changes in the environmental conditions composition and activity of microbiota and dysbiosis. Obviously, this did not happen in our *in vitro* fermentation model because host factors are not involved.

Enumerating specific bacterial groups by PMA-qPCR provided first a global information on the composition of the swine gut microbial community. Rifampicin induced important changes in *Bacteroides*, *Lactobacillaceae* and total 16S rRNA gene contents at the end of the colonic fermentation while no major effects were observed when MccJ25 and reuterin were added separately. Furthermore, 16S rRNA gene sequencing using the Illumina MiSeq approach revealed that rifampicin significantly impacted microbiota diversity as compared to the tested antimicrobials in the PolyFermS model. Taxonomically, rifampicin was shown to induce significant changes in bacterial abundances when compared to the other treatment groups, which results in a significant increase of the relative abundance of *Proteobacteria* and *Firmicutes* phyla. In published studies on swine fecal and colonic microbiomes *Firmicutes*, *Bacteroidetes* and *Proteobacteria* have been reported as the most abundant phyla (Poroyko et al., 2010; Holman et al., 2017). These prevalences are in accordance with our findings shown in Figure 4. Moreover, the present meta-analysis of a porcine intestinal bacterial community has revealed the predominance of some bacterial families in both test reactors, such as *Ruminococcaceae*, *Prevotellaceae* and *Muribaculaceae*, and some unidentified members of the genus *Prevotella*, *Succinivibrio* and *Escherichia-Shigella*. Similar results have been observed in previous studies using laboratory models of the colon based on culture in liquid media (Le Blay et al., 2008; Van den Abbeele et al., 2010; Tanner et al., 2014). *Prevotella* has also been shown to be the most abundant bacterial group in the porcine colon (Kim et al., 2011; Looft et al., 2012). This is likely related to the typical swine diet, which is relatively rich in polysaccharides requiring fibrolytic bacteria for their breakdown in the colon to provide SCFAs as an energy source for other members of the microbial community (Vital et al., 2014). Species of *Prevotella* reportedly produce acetate, which is an essential SCFA for butyrate production by some bacterial species in the swine gut ecosystem (Looft et al., 2014). Furthermore, we observed significant variations in the relative abundance of some bacterial groups, especially with rifampicin, which induced an important increase in the prevalence of *Escherichia-Shigella* genera in the middle of the fermentation (Figure 5). Such variations were not observed following addition of MccJ25 or reuterin to the bioreactors. They might be due to the broad-spectrum activity of rifampicin that affects both Gram-positive and Gram-negative bacteria, unlike the narrow spectrum of MccJ25, which targets bacteria belonging to the *Enterobacteriaceae* family only. However, reuterin, which was expected to have a broad spectrum of action (Cleusix et al., 2007; Stevens et al., 2011), did not show significant changes in this bacterial groups. This could be potentially due to the different mechanisms subtending the activities of MccJ25 and reuterin. This has been observed in our previous study, which showed that reuterin did not inhibit *Salmonella* Newport in the PolyFermS model (Naimi et al., 2020). The addition of reuterin (1.3 mM) was previously reported to significantly and selectively decrease *E. coli* without affecting other bacterial populations in an *in vitro* colonic fermentation model mimicking the proximal adult colon, inoculated with immobilized fecal microbiota (Cleusix et al., 2008). Indeed, we have mentioned that the lack of inhibitory activity of reuterin could be due to non-specific interactions with various compounds in the modified Macfarlane medium, or possibly to instability under these conditions. But, reuterin is an antimicrobial multicomponent system consisting of 3-hydroxy-propionaldehyde (3-HPA), its dimer and hydrate, and acrolein which has been suggested to be the active component of the system (Engels et al., 2016). Moreover, the equilibrium between acrolein and 3-HPA is dynamic and highly dependent on temperature and storage duration (Asare et al., 2018, 2020). This complex, subtle and dynamic equilibrium could also explain both the lack of anti-*Salmonella* activity and the absence of perturbations of the microbiota provoked by reuterin in the Polyferm model.

Similar results were observed when analyzing the impact of these antimicrobial compounds on metabolic activity of the swine colonic microbiota. Indeed, the LC-MS method developed as a complement to microbial diversity analysis provided important clarification of the effects of natural antimicrobials on the microbial ecosystem of the swine colon. As expected, LC-MS analysis showed that reuterin had no impact on the metabolome compared to the control group. However, MccJ25 appeared to significantly affect the metabolome and was shown distinguishable from other treatment groups, as shown by the LC-MS chromatograms. The approach of molecular networking based on LC-MS/MS profiling allowed to observe and identify degradation forms of MccJ25 in addition to the native microcin, thus forming that we defined as the “MccJ25 degradome”. Such a type of analysis had been previously performed in one of our previous studies that described the degradation products of MccJ25 under gastro-intestinal (GI) conditions (Naimi et al., 2018). In the present study, MS/MS spectra of MccJ25 in swine colonic conditions have shown two degraded forms with no cleaved segments. The first one is located at the G12-I13 peptide bond, while the second one is located at G14-T15, both being in the loop portion of the lasso structure, which is more exposed to the environment. Degradome analysis revealed other degradation products of MccJ25 involving cleavages in addition to the loss of two, four or five residues. These results confirm the partial degradation of MccJ25 under GI conditions due to pancreatic proteases (Naimi et al., 2018). This degradation pattern further validates that MccJ25 is unlikely to affect persistently the GI tract and therefore suggests that it should be considered as a safe alternative to antibiotics and for food applications.

Interestingly, metabolic profiling analysis has demonstrated an impact of rifampicin on the metabolome of the swine colonic microbiota. In this study, molecular network based on MS/MS analysis showed a clear clustering structure corresponding to the intact form of the antibiotic as well as its different degradation products. These metabolites, identified as demethyl rifampicin, desacetyl rifampicin and mono-oxygenated rifampicin, have been observed in swine colonic fermentation samples by LC-MS/MS. Such metabolites were also identified previously *in vitro* using an LC-MS approach that characterized the biotransformation products of rifampicin after incubation with a rat liver fraction (Prasad and Singh, 2009). Similar degradation metabolites were also generated *in vivo* by administering rifampicin to rats (Prasad and Singh, 2009). Rifampicin biotransformation by the fungus *Cunninghamella elegans* was also studied by UHPLC-MS and the degradation products appeared to impact the microbiota diversity and affect the metabolome (Sponchiado et al., 2020), in agreement with our present data.

Since MccJ25 appears to be a potent inhibitor of *Salmonella* Newport in the PolyFermS continuous culture model of the porcine proximal colonic microbiome as shown previously (Naimi et al., 2020), it should be considered as a potential alternative to antibiotics in pork production. In this study, MccJ25 does not appear to affect the composition of this bacterial community, as demonstrated by PMA-qPCR and 16S rRNA MiSeq sequencing analysis. This is important since it has been shown that changes in gut microbiota composition are associated with various metabolic disorders and likely subject the animal to stress in any event. LC-MS data analysis and metabolomic profiling allowed to demonstrate that MccJ25 induces only subtle changes in both the microbial diversity and the metabolome of the swine colon microbiota. Thus, MccJ25 is better than reuterin which does not perturb the microbiota but does not inhibit neither the growth of *Salmonella* Newport, and better than rifampicin which inhibits *Salmonella* growth but induces significant modification in amino acid levels.

## Conclusion

Taking into account its pathogen-selective and potent inhibitory activity, its stability due to its compact and stable structure and the only subtle changes it induces in the microbiota composition, MccJ25 might be considered as a possible alternative to antibiotics in pig farming. Although the PolyfermS pig microbiota fermentation model does not mimic host factors and the treatments were only applied for 24 h, the present study affords a first proof of concept of a possible use of MccJ25 in pig farming. Future studies should be focused on validating *in vivo* the effectiveness of MccJ25 as an inhibitor of *Salmonella* in farmed pigs as well as evaluating its impact on the intestinal microbiota.

## Data availability statement

The 16S rRNA sequencing data presented in this study can be found in the online repository European Nucleotide Archive (ENA) https://www.ebi.ac.uk/ena/ under the accession number PRJEB52762. The LC-MS metabolomics data generated and analyzed in this manuscript are available on UCSD Metabolomics Workbench https://www.metabolomicsworkbench.org/ under the mwTab Identifier: severinezirah_20211113_140942.

## Author contributions

SN designed and performed experiments, carried out data analysis, and wrote the manuscript. IF managed the overall project, participated in the design of the experiments, analysis and interpretation of data, and wrote the manuscript. SR contributed to manage the overall project, helped for the interpretation of data, and wrote the manuscript. SZ participated for the design of the experiments, performed metabolomic analysis, helped for analysis and interpretation of data, and wrote the manuscript. AG analyzed the 16S sequencing data, helped in interpretation of data, and wrote the manuscript. CL helped for the interpretation of data and wrote the manuscript. All authors approved the final version of the manuscript.

## Footnotes

1
https://zenodo.org/record/6423397#.Yr9DzuzMK3I

2
https://zenodo.org/record/6473394#.Yr9JjuzMK3I

3
www.r-project.org

## Abbreviations

MccJ25: microcin J25
IR: inoculum reactor
TR1: test reactor 1
TR2: test reactor 2
16S rRNA: 16S ribosomal ribonucleic acid
LC-MS: liquid chromatography coupled to mass spectrometry
PCA: principal component analysis.
