# Supplementary material for: Impact of microcin J25 on the porcine microbiome in a continuous culture model
Source: Front Microbiol. 2022 Aug 3;13:930392. doi: 10.3389/fmicb.2022.930392 (PMC9383034; doi:10.3389/fmicb.2022.930392)
Supplement: Supplementary file 1 [file Data_Sheet_2.docx]

Supplementary Material

Impact of microcin J25 on the porcine microbiome in a continuous culture model

Sabrine Naimi, Séverine Zirah, Anna Greppi, Christophe Lacroix, Sylvie Rebuffat, and Ismail Fliss^*^

*** Correspondence:** Corresponding Author: [ismail.fliss@fsaa.ulaval.ca](mailto:ismail.fliss@fsaa.ulaval.ca)

# Supplementary Figures and Tables

## Supplementary Tables

**Table S1.** Primers used for enumerations of swine colonic bacteria and *Salmonella* Newport by the PMA-qPCR method.

| **Target** | **Bacterial strain** | **Primer** | **Sequence 5’-3’** | **Reference** |
| --- | --- | --- | --- | --- |
| Total 16S rRNA genes | *E. coli* ATCC25922 | Eub338F | ACT CCT ACG GGA GGC AGC AG | Guo *el al*. 2008 ^1^ |
|  |  | Eub518R | ATT ACC GCG GCT GCT GG |  |
| *Bacteroides* | *B. thetaiotaomicron* ATCC29741 | Bac303F | GAA GGT CCC CCA CAT TG | Ramirez-Farias *et al*. 2008 ^2^ |
|  |  | Bfr-Fmrev | CGC KAC TTG GCT GGT TCA G |  |
| *Lactobacillaceae* | *L. acidophilus* ATCC 4356 | F_Lactos 05 | AGC AGT AGG GAA TCT TCC A | Furet et al. 2009 ^3^ |
|  |  | R_Lacto 04 | CGC CAC TGG TGT TCY TCC ATA TA |  |
| *Enterobacteriaceae* | *E. coli* ATCC25922 | Eco1457F | CAT TGA CGT TAC CCG CAG AAG AAG C | Bartosch et al. 2004 ^4^ |
|  |  | Eco1652R | CTC TAC GAG ACT CAA GCT TGC |  |
| *Salmonella* | *Salmonella* Newport ATCC 6962 | invAF | CGTTTCCTGCGGTACTGTTAATT | Li & Chen. 2013 ^5^ |
|  |  | invAR | TCGCCAATAACGAATTGCCCGAAC |  |

**Table S2. Degradome of MccJ25 in the swine colonic fermentation model.** Ions detected for the intact molecules and main degradation products, identified by LC-MS/MS and molecular networking (experimental and calculated monoisotopic *m/z* and retention time, RT).

| **Molecule** | **Raw formula** | **Ions** | **Exp. *m/z*** | **Calc. *m/z*** | **RT (min)** |
| --- | --- | --- | --- | --- | --- |
| **MccJ25** | **C_101_H_139_N_23_O_27_** | **[M+2H]^2+^**  **[M+3H]^3+^** | **1054.0146**  **703.0141** | **1054.0178**  **703.0143** | **10.8** |
|  | C_101_H_141_N_23_O_28_ | [M+2H]^2+^  [M+3H]^3+^ | 1063.0221  709.0180 | 1063.0231  709.0178 | 9.7 |
|  | C_101_H_141_N_23_O_28_ | [M+2H]^2+^  [M+3H]^3+^ | 1063.0214  709.0177 | 1063.0231  709.0178 | 9.9 |
|  | C_93_H_127_N_21_O_26_ | [M+2H]^2+^  [M+3H]^3+^ | 977.9690  652.3160 | 977.9703  652.3160 | 9.6 |
|  | C_86_H_115_N_19_O_24_ | [M+2H]^2+^  [M+3H]^3+^ | 899.9245  600.2860 | 899.9254  600.2860 | 9.5 |
|  | C_82_H_108_N_18_O_22_ | [M+2H]^2+^  [M+3H]^3+^ | 849.4012  566.6036 | 849.4016  566.6035 | 9.5 |

**Table S3. Degradome of rifampicin in the swine colonic fermentation model.** Ions detected for the intact molecules and main degradation products, identified by LC-MS/MS and molecular networking (experimental and calculated monoisotopic *m/z* and retention time, RT).

| **Molecule** | **Raw formula** | **Ions** | **Exp. *m/z*** | **Calc. *m/z*** | **RT (min)** |
| --- | --- | --- | --- | --- | --- |
| **Rifampicin**   | **C_43_H_58_N_4_O_12_** | **[M+H]^+^** | **823.4110** | **823.4124** | **12.0** |
| Demethyl rifampicin | C_42_H_56_N_4_O_12_ | [M+H]^+^ | 809.3973 | 809.3968 | 11.4 |
| Desacetyl rifampicin | C_41_H_56_N_4_O_11_ | [M+H]^+^ | 781.4016 | 781.4018 | 11.4 |
| Mono-oxygenated rifampicin | C_43_H_58_N_4_O_13_ | [M+H]^+^ | 839.4071 | 839.4073 | 11.3  12.3 |

## Supplementary Figures

**A**

**C**

**B**

**D**

**Figure S1.** Mean concentration (log10 copy number per mL of effluent) of specific bacterial groups measured by PMA-qPCR in PolyFermS model test reactors TR1 and TR2 for 24 h after adding **(A)** *Salmonella* Newport (Salmo) at an initial concentration of 10^7^ cfu mL^−1^, **(B)** reuterin + *Salmonella* Newport (Reut), **(C)** rifampicin + *Salmonella* Newport (Rifa), **(D)** MccJ25 + *Salmonella* Newport (J25). Bars indicate standard deviation resulting from means of two independent repetitions in TR1 and TR2.


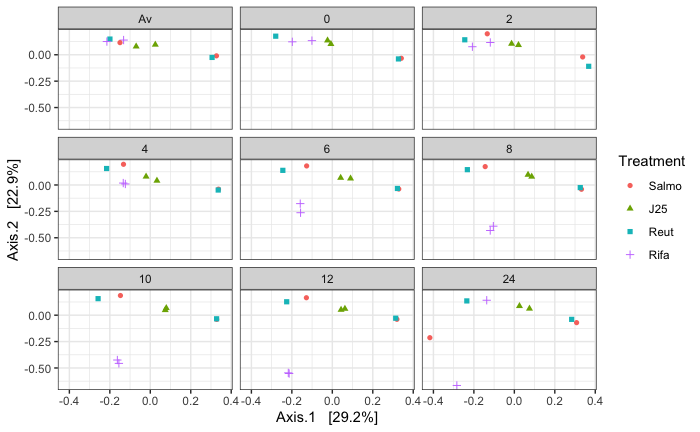


**Figure S2.** Principal coordinate analysis (PCoA) of porcine microbiota after inoculation of *Salmonella* Newport alone (Salmo) and in combination with MccJ25 (J25), reuterin (Reut) or rifampicin (Rifa) *in vitro,* based on Bray-Curtis similarity matrix for each time points from the two independent experiments. Dots are colored by treatment.


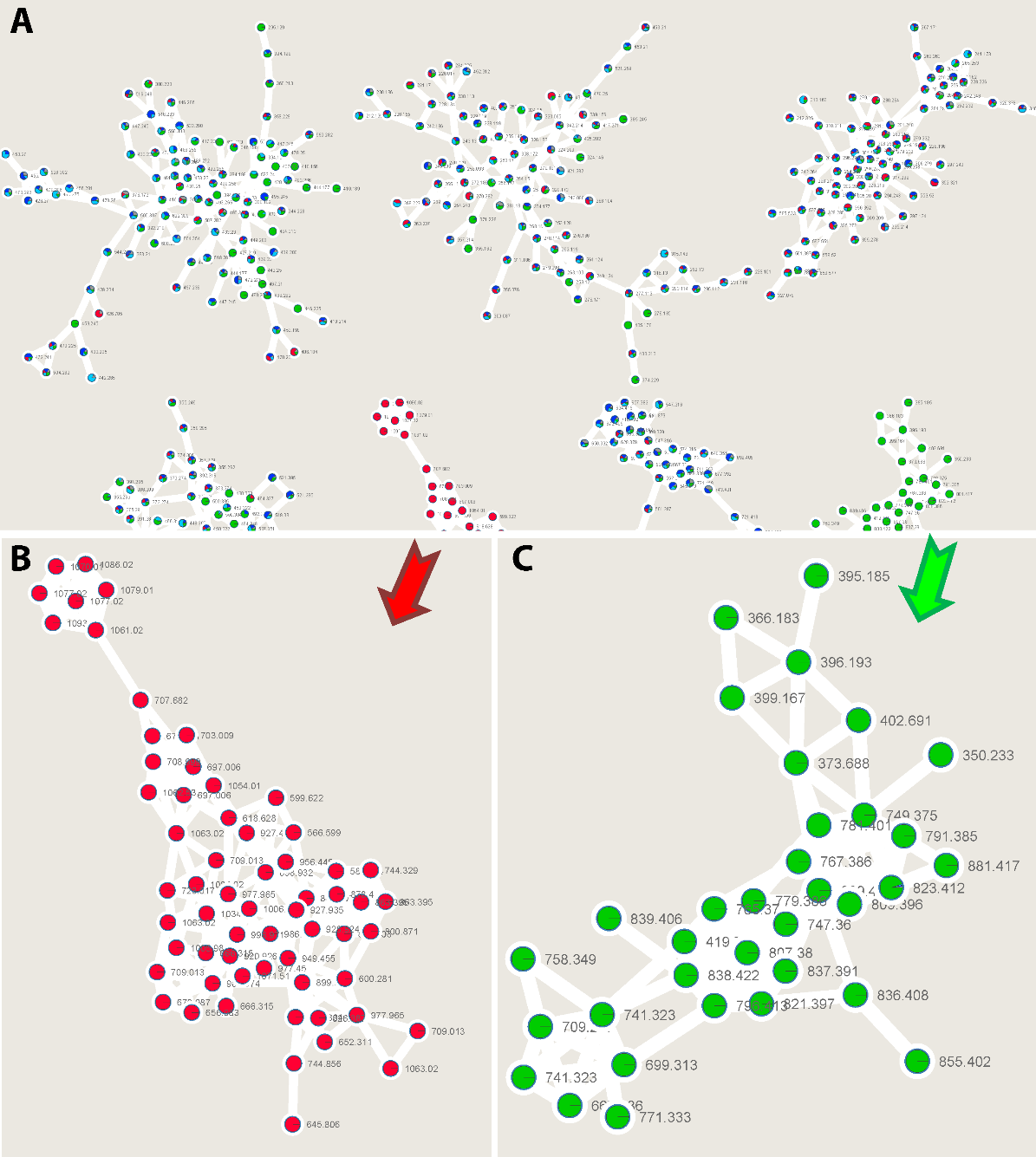


**Figure S3. Molecular network constructed from LC-MS/MS data on the metabolomic extracts of swine colonic microbiota in the PolyFermS.** A. Upper part of the whole network. B Clusters assigned to MccJ25 only, showing the intact peptide and its degradation products. C. Cluster assigned to rifampicin only showing the intact antibiotic and its metabolites.

|  | 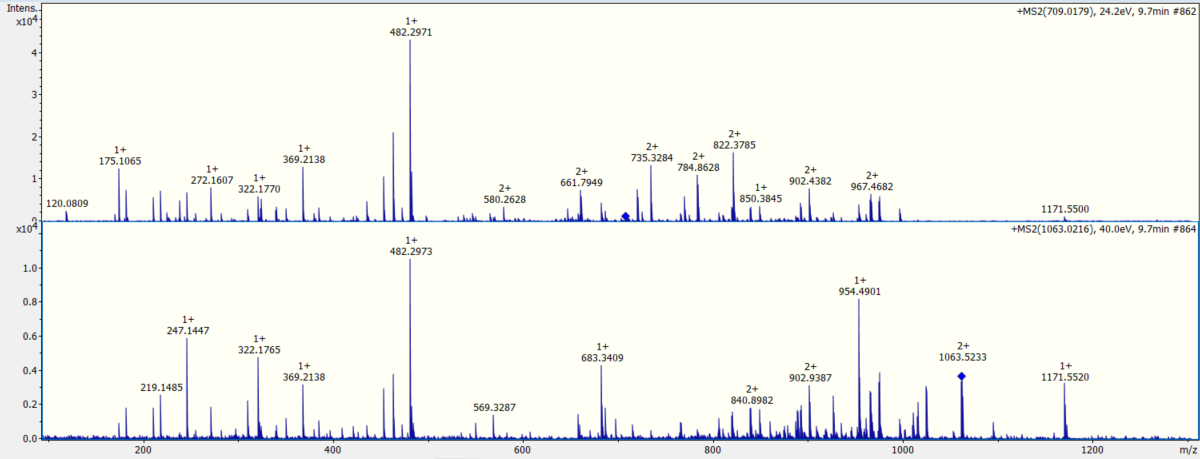  B  A |
| --- | --- |

**Figure S4. MS/MS spectra of MccJ25 hydrolyzed at G12-I13 formed in the PolyFermS**. A: [M+3H]^3+^ (m/z 709.02, CE 24.2 V), B: [M+2H]^2+^ (m/z 1063.02, CE 40 V). The hydrolysis site was determined from the + 18 u increment product ions, as already reported ^6^.

|  | 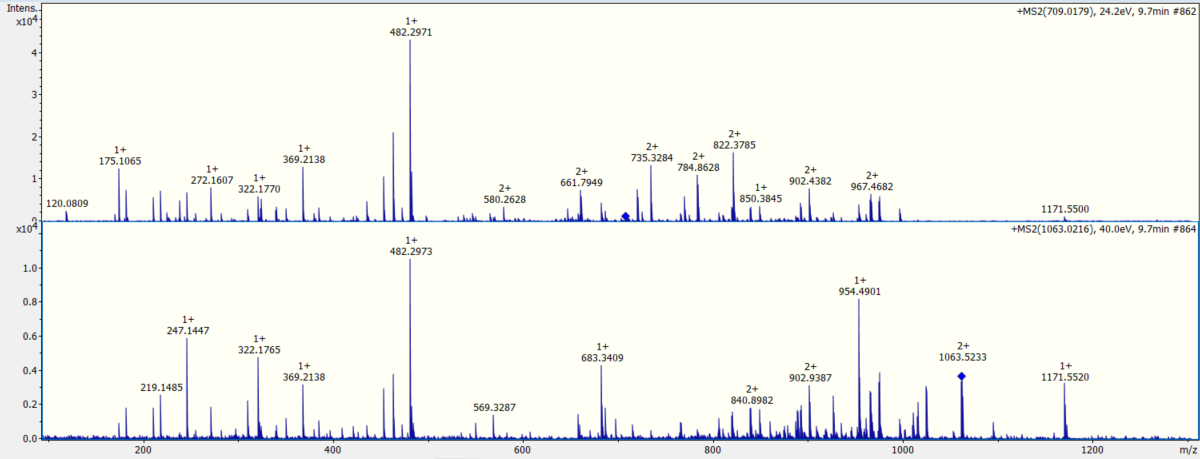 |
| --- | --- |

**Figure S5. MS/MS spectra of MccJ25 hydrolyzed at G14-T15 formed in the PolyFermS**. A: [M+3H]^3+^ (m/z 709.02, CE 24.2 V), B: [M+2H]^2+^ (m/z 1063.02, CE 40 V). The hydrolysis site was determined from the + 18 u increment product ions, as already reported ^6^.

# References

1. Guo X, Xia X, Tang R, Zhou J, Zhao H, Wang K. Development of a real‐time PCR method for Firmicutes and Bacteroidetes in faeces and its application to quantify intestinal population of obese and lean pigs. Lett Appl Microbiol 2008; 47:367–73.

2. Ramirez-Farias C, Slezak K, Fuller Z, Duncan A, Holtrop G, Louis P. Effect of inulin on the human gut microbiota: stimulation of Bifidobacterium adolescentis and Faecalibacterium prausnitzii. Br J Nutr 2008; 101:541–50.

3. Furet J-P, Firmesse O, Gourmelon M, Bridonneau C, Tap J, Mondot S, Doré J, Corthier G. Comparative assessment of human and farm animal faecal microbiota using real-time quantitative PCR. FEMS Microbiol Ecol 2009; 68:351–62.

4. Bartosch S, Fite A, Macfarlane GT, McMurdo ME. Characterization of bacterial communities in feces from healthy elderly volunteers and hospitalized elderly patients by using real-time PCR and effects of antibiotic treatment on the fecal microbiota. Appl Environ Microbiol 2004; 70:3575–81.

5. Li B, Chen J-Q. Development of a sensitive and specific qPCR assay in conjunction with propidium monoazide for enhanced detection of live Salmonella spp. in food. Bmc Microbiol 2013; 13:273.

6. Naimi S, Zirah S, Hammami R, Fernandez B, Rebuffat S, Fliss I. Fate and biological activity of the antimicrobial lasso peptide microcin J25 under gastrointestinal tract conditions. Front Microbiol 2018 ; 9 :1764.
